# Prolong psychosis preceding cognitive and motor symptoms; an unusual presentation in Huntington's disease

**DOI:** 10.1192/bjo.2021.341

**Published:** 2021-06-18

**Authors:** Nida Khan, Marium Mansoor

**Affiliations:** The Aga Khan University Hospital

## Abstract

**Objective:**

To describe an unusual clinical presentation of Huntington's disease.

**Case report:**

A 39-years-old married female, a homemaker, presented to the psychiatry clinic with her husband, with over a nine-year history of decreased sleep, suspiciousness, self-talking, agitation, anger outbursts, aggression, and social isolation. The patient was diagnosed with schizophrenia. Previously, she received various antipsychotics and Electroconvulsive therapy (ECT). The patient showed a partial response to treatment. Over the last 2-3 years, the patient had a progressive decline and later required supervision in her Activities of Daily Living (ADLs). She developed slurred speech limited to 1-2 worded answers, gait disturbance, falls, involuntary movements of the trunk and distal extremities, bowel and bladder incontinence, and severe weight loss. The patient's mother and older brother had a history of death in their early 40s due to an unknown cause.

At presentation, the patient was restless, irritable, self-talking incoherently, neither made nor maintained eye contact and tried hitting and biting upon approaching closely. She did not respond to any queries or followed commands. The patient showed poor personal hygiene. On examination, the patient was hemodynamically stable, had a loss of muscle bulk, broad-based gait, and choreiform movements of the trunk and distal extremities. We admitted the patient to the psychiatry ward and also consulted the neurology team. Her blood investigations showed ASMA antibodies positive, MRI brain was suggestive of Huntington's disease (HD), and her genetic test for Huntington gene confirmed the diagnosis of HD. We started the patient on Fluoxetine, Clonazepam, and Olanzapine. The patient showed a decrease in agitation, and her self-talking stopped.

**Discussion:**

HD is a rare genetic disease that has well-characterized symptoms. However, as seen in our patient, these symptoms can evolve and progress unusually in the early and middle stages. Psychosis in HD patients is rare but known. Psychosis is rare in HD and usually presents after a clear clinical picture of HD is apparent. Our case discussed psychotic symptoms in the pre-choreic stage of HD which adds to the existing evidence on challenging presentations and management of HD. Further research can help increase confidence in these outcomes and treatment guidelines.

**Conclusion:**

Our case highlights an unusual clinical presentation of HD, which can be challenging and lead to diagnostic delays. We recommend a thorough approach to history and revision of diagnosis in case of atypical presentations.

